# Health professionals identify components of the International Classification of Functioning, Disability and Health (ICF) in questionnaires for the upper limb

**DOI:** 10.1590/bjpt-rbf.2014.0135

**Published:** 2016-01-19

**Authors:** Stella V. Philbois, Jaqueline Martins, Cesário S. Souza, Rosana F. Sampaio, Anamaria S. Oliveira

**Affiliations:** 1 Faculdade de Medicina de Ribeirão Preto, Universidade de São Paulo (USP), Ribeirão Preto, SP, Brazil; 2 Escola de Educação Física, Fisioterapia e Terapia Ocupacional, Universidade Federal de Minas Gerais (UFMG), Belo Horizonte, MG, Brazil

**Keywords:** questionnaires, shoulder, international classification of functioning, disability and health, rehabilitation, health care

## Abstract

**BACKGROUND:**

Several Brazilian studies have addressed the International Classification of Functioning, Disability and Health (ICF), but few have analyzed the knowledge of the health professionals with regards to the ICF.

**OBJECTIVE:**

To verify whether the classification of the items in the Brazilian-Portuguese versions of The Shoulder Pain and Disability Index (SPADI) and The Disabilities Arm, Shoulder and Hand (DASH) questionnaires, obtained from health professionals who worked with patients having upper limb injuries, could be related to ICF components as defined by others studies.

**METHOD:**

There were 4 participants for the group "professionals with high familiarity of the ICF (PHF)" and 19 for the group of "professionals with some or no familiarity of the ICF (PSNF)". The participants judged whether the items on the two questionnaires belonged to the ICF body function, body structure or activity-participation component, and marked a confidence level for each trial using a numerical scale ranging from zero to 10. The items were classified by the discriminant content validity method using the Student's*t*-test and the Hochberg correction. The ratings were compared to the literature by the percentage of agreement and Kappa coefficient.

**RESULTS:**

The percentage of agreement of the rating from the PSNF and the PHF groups with the literature was equal to or greater than 77%. For the DASH, the agreement of the PSNF and PHF groups with the literature were, respectively, moderate (Kappa=0.46 to 0.48) and substantial (Kappa=0.62 to 0.70).

**CONCLUSIONS:**

Health professionals were able to correlate the three components of the ICF for most items on the 2 questionnaires, demonstrating some ease of understanding the ICF components. However, the relation of concept of pain with body function component is not clear for professional and deserves a more attentive approach.

## Introduction

The International Classification of Functioning, Disability and Health (ICF) represents a perspective on incapacity and disability based on the biopsychosocial model of health care^1^. The ICF describes functionality and individual disability in relation to body function component (F), body structure (S) and activity and participation (AP), which are influenced by environmental and personal contextual factors^1^.

In Brazil, studies on the ICF involved research about its conceptualization^2^^,^ 3, reviews of its application^4^^,^ 5 and the *Core Sets* 6, epidemiological studies^7^^,^ 8 and use public policies^9^ and training^10^^-^ 12, with emphasis on the areas of neurology and orthopedics^4^. Some of these studies have sought to establish the relationship between different questionnaires and the components of the ICF^13^^-^ 15 or its *Core Sets* 16 ^,^ 17. Others either evaluated the functional profile of neurological^18^^-^ 20, oncological and geriatric^21^^,^ 22 patients through the ICF or validated questionnaires for the classification of the components, or studied the effects of interventions on patients^23^ or validated*Core Sets* for neurological^24^, rheumatological^25^ and orthpedic^26^^,^ 27 patients.

It is noteworthy that among the research developed using the ICF in Brazil; only one study evaluated the understanding of the ICF in the educational context of graduate students^11^. Silva et al.^11^ concluded that physical therapy students in the orthopedics area were unfamiliar with the ICF components because their assessments were based on the biomedical model, centered on the pathology and on the body structures and functions. This unfamiliarity may be a common reality to other health professionals as well.

Ruaro et al.^4^ argued that expanding the use of the ICF could be achieved through the training of academics and professionals. However, studies investigating health professionals who had not received previous training, which aimed to analyze the ability of a professional to recognize the ICF components in assessment tools commonly used in their practice, are lacking. This assertion justifies the present study which the authors hoped could contribute to the implementation of the ICF by revealing the easiness and difficulties in identifying the ICF components: important aspects that should be considered in the training of health professionals.

Besides, there are some difficulties when applying the ICF in clinical settings^5^ since it is dependent on developing evaluations that include the components of the ICF or on the availability of validated questionnaires for ICF^28^^-^ 32. Thus, one standardized way of assessing the applicability of the ICF components by various health professionals would be to examine how these professionals relate the components of the ICF classification with outcome measure questionnaires commonly used in the clinic. This process of connecting outcome measures to the ICF classification has been called*linking* 28 ^-^ 32.

Considering the area of orthopedic rehabilitation, some questionnaires, commonly used in clinic situations, have been linked to the ICF^30^^-^ 32. Among these are The Shoulder Pain and Disability Index (SPADI)^33^, which evaluates shoulder pain and disability, and The Disabilities of the Arm, Shoulder and Hand (DASH)^34^, which assesses symptoms and physical, psychological and social functions of individuals with upper limb dysfunction.

Therefore, the objective of this study was to determine whether the classification of the questionnaires SPADI, and DASH to 3 components of the ICF, when completed by health professionals who do research or treat and rehabilitate upper limb injuries, and have different levels of knowledge of the ICF, agree with the classification of the SPADI as presented by Roe et al.^30^ and the DASH as presented by Dixon et al.^31^ and Drummond et al.^32^.

The study hypothesis was that health professionals having less familiarity with the ICF would have greater difficulty correlating the items in 2 shoulder questionnaires with the ICF classifications than those health professionals who have greater familiarity with the ICF components, when comparing to literature. It was hoped the results of this study might contribute to a better understanding of how the components of the ICF classification are recognized by health professionals with different levels of knowledge of the ICF and highlight concepts from the 2 shoulder questionnaires or ICF components that require more attention.

## Method

### Participants

Twenty-nine participants were recruited for this cross-sectional, observational study. The participants were contacted by telephone or directly, at public hospitals and private clinics of a city of state of São Paulo or public institutions of higher education from the southern region of Brazil.

The participants were divided into two groups according to their degree of familiarity with the ICF. Four participants composed the group of "professionals with high familiarity (PHF)" with the ICF, and 19 participants composed the group of "professionals with some or no familiarity (PSNF)" with the ICF.

The PHF group had a sample size (4) and profile of participants similar to studies investigating the content validity of several questionnaires, which had sample sizes varying from two to 20^35^^,^ 36 and, about ICF, had participants with good knowledge of its components and taxonomies^30^^,^ 32 ^,^ 37. The authors considered the relatively small sample size of the PHF group to be acceptable, due the limited number of professionals in Brazil with this profile, and resulted in the asymmetry between the groups.

The inclusion criteria for the PHF group were being a clinician or researcher involved in the treatment or research of musculoskeletal or orthopedic rehabilitation of the upper limb and were working or had read publications about the ICF in scientific journals, which was considered sufficient proof of their in-depth knowledge of the ICF.

For the PSNF group, inclusion criteria were "being a clinician or researcher involved in the treatment or research of musculoskeletal or orthopedic rehabilitation of the upper limb and who had no specific knowledge of the ICF. This means participants were not conducting research involving the ICF and had a level of knowledge equal to or less than five points on the numerical rating scale, where 10 points indicated that the participant fully understood the ICF and 0 indicated no knowledge of the ICF. All participants who agreed to participate in the study signed an Informed Consent form approved by the Ethics Committee of the Clinical University Hospital of the Faculdade de Medicina de Ribeirão Preto, Universidade de São Paulo (USP), Ribeirão Preto, SP, Brazil (Protocol n° 8857/2013).

### Instruments

The SPADI and DASH questionnaires, are often used for functional assessment of shoulder dysfunctions, and are among the 10 questionnaires most cited in the literature^30^. Both questionnaires, including their Brazilian versions, had their psychometric properties tested^38^^-^ 42.

The SPADI-Brazil^39^ questionnaire has 13 items, five of which assess pain, and eight of which assess disability. Items were scored on a numerical rating scale of 10 points, with zero indicating no pain/no difficulty and 10 indicating severe pain/could not perform the activity. The scores ranged from 0 to 100 for each domain and the higher the score the more severe was the injury to the patient's shoulder.

The Brazilian version of the DASH^40^evaluates various dysfunctions of the upper limb, having 30 items, 21 of which evaluates physical function, six assess symptoms, two evaluates social functions and one evaluates psychological functions. The items were scored on a Likert scale ranging from zero to five points and the total score ranged from 0 to 100, with the maximum score indicating a lower quality of life.

The material available to the participants contained instructions and items from the SPADI and DASH questionnaires distributed randomly. Each item had three options defined by the ICF components: body function, body structure and activity-participation. For each item, the participants provided a Yes/No answer and gave a confidence percentage rating for each judgement on a numerical rating scale ranging from 0% to 100% in increments of 10%^31^. The activity-participation component was also tested to discriminate whether the questionnaire item referred to an activity itself or to participation in the activity.

Definitions of the three components of the ICF^1^ were included at the top of each page, and at the end of the material, questions about the descriptive data of the sample were presented. The material omitted the identification of the participants, but posed the question "What is your level of familiarity with the ICF?" to distinguish the PHF and PSNF groups. The response options were "High familiarity" or "Some or no familiarity". The participants also answered questions about their level of knowledge of the ICF through a numerical rating scale ranging from 0 to 10, where zero indicated that the participant was unfamiliar with the ICF and 10 indicated a full understanding of the ICF.

### Procedures

Participants interested in participating in the study received an email with information related to the study and with the informed consent form. After returning the signed informed consent form, each participant received the materials by post or email and had one week to return the material. To ensure the anonymity of the participants, the material returned by mail or electronically was received by a third party not linked to the research and delivered to the researchers without identification.

Participants were asked to make an individual judgment on the items without discussing their answers with any other individual, whether those other individuals were involved in the study or not. Thus, participants had to make their judgment based only on prior knowledge of the classification or on the brief definitions presented by the material.

Each item was rated three times. First, participants had to check if the item was related to the ICF component or not, and then mark the confidence level for the judgment of each response using the numerical rating scale ranging from 0-100%^31^. Thus, 43 items were rated from the 2 shoulder questionnaires, totaling 129 judgments. Finally, each participant had to respond to questions descriptively and answer questions regarding their level of knowledge of the classification.

### Statistical analysis

The descriptive analyses were presented as mean and standard deviation for the quantitative data and relative frequency for the qualitative data. The classifications of the items, according to the ICF components chosen by the participants, were analyzed with discriminant content validity and the Student's*t*-test^31^^,^ 37. In addition to establishing the content validity of the items of the questionnaire, the method also established whether the three theoretical components of the ICF could be measured discriminately from one another^31^^,^ 37. Thus, the discriminative power of the test was the result of the ratings assigned by the participants, relating each item of the two questionnaires to the components of the ICF^31^^,^ 37.

The judgments of each item were scored as 1 when the professional judged that the item was related to the ICF component (marking YES) and -1 when unrelated to the ICF component (marking NO). This value was then multiplied by the level of confidence of each rating, expressed as an absolute percentage, and varied from 0 to 1. Thus, the final value obtained for each judgment ranged from -1 to +1. Data that were lost or unmarked were scored as zero^31^.

The classification of the items in one of the seven possible categories - body function (F), body structure (S), activity and participation (AP), body function and body structure (F.S), body function and activity and participation (F.AP), body structure and activity and participation (S.AP) and all of them (F.S.AP) was conducted using the one sample Student's *t*-test. The item was classified as related to the ICF component when the judgment given was significantly greater than zero. The Hochberg^43^ correction for multiple comparisons was performed and all procedures were conducted using the software R Core Team^44^.

The classification of items from the SPADI questionnaire was compared to the study by Roe et al.^30^, who associated the items to body function or activity-participation components of the ICF. The classification of the items from the DASH was compared to the study by Dixon et al.^31^ and Drummond et al.^32^ which classified each item specifically. The domains of physical and social function of the DASH should relate to the activity-participation component. The domain of symptoms for function and/or body structure components and item 30 of the psychological function were not considered to represent a personal factor and thus, was not classified in the study^31^^,^ 32.

The agreement between the classification obtained by the PHF and PSNF groups with the literature^30^^-^ 32 were conducted with the simple percentage of agreement and the Kappa coefficient with a 95% confidence interval^45^. The agreement by the Kappa coefficient was classified as follows: poor if < 0; weak if between 0.01 and 0.20; light if between 0.21 to 0.40; moderate if between 0.41 to 0.60; substantial if between 0.61 and 0.80; almost perfect if between 0.81 to 0.99 and perfect if 1^45^. In addition, if the groups could discriminate in activity or participation, the items judged as an activity-participation component were also described as performed by Dixon et al.^31^.

## Results

The study contacted 29 health professionals, but one refused to participate because the subject was worry about the lack of blinding. The material was sent to 28 participants and 23 returned the material. It wasn't possible to know why the subjects didn't return the material, because the researchers were blinded about assessments. Thus, data of 23 participants were analyzed and the descriptive data is presented in Table 1.

**Table 1:** - Descriptive data of the participants in the group of "professionals with high familiarity (PHF)" (n=4), and the group of "professionals with some or no familiarity (PSNF)" (n=19) with the International Classification of Functioning, Disability and Health (ICF).

Characteristics	Values
Time after graduation Mean (year)±SD	11.9±9.9
Undergraduate program (%) Physical Therapy/Occupational Therapy Medicine – Orthopedics and Physiatrist	17 (73.9%) / 3 (13.0%) 3 (13.0%)
Profession (%) Professor Graduate student of physical therapy Physical therapist/Occupational therapist Physician	9 (39.1%) 2 (8.7%) 9 (39.1%) / 1 (4.3%) 2 (8.7%)
How long do you work treating the upper limb? Mean (year)±DP	8.5±7.6
Do you use questionnaires regularly? (%) Yes / No	18 (78.3%) / 5 (21.7%)
Do you know or had heard about the ICF? (%) Yes / No	21 (90.9%) / 2 (9.1%)
How did you know or hear about the ICF? (%) Courses/Lecture/Site Post-graduation/Academic community Work with people who use the ICF	10 (43.5%) 10 (43.5%) 5 (21.7%)
Do you consider the ICF important? (%) Yes / Don’t know the ICF	19 (82.6%) / 4 (17.4%)
Familiarity level to the ICF (%) Professionals with high familiarity to ICF (PHF) Professionals with some or no familiarity to ICF (PSNF) - Do not use ICF at work - Use ICF at work - No answer	4 (17.4%) 19 (82.6%) 14 (73.7%) 3 (15.8%) 2 (10.5%)
How do you know the ICF on a scale of 0 to 10? Professionals with high familiarity to ICF (PHF) Professionals with some or no familiarity to ICF (PSNF)	Mean±SD 9.25±1.8 3.2±2.2

SD: standard deviation.

The power of the test was analyzed with the Student's *t*-test in relation to the component with which the item was correlated, and for the undefined items, the three components were considered for the power analysis. For the PSNF group, the power values were above 0.80 (beta=0.20) on 76.92% of the items of the SPADI and on 83.33% of the items of the DASH, considering an alpha of 0.05. The power of the test was not conducted for the PHF group because the sample size was small and the items judged did not present a standard deviation. However, the sample size differences between groups did not affect the analyses that were conducted only within group.

The percentage of agreement values was relatively high (above 77%), but lower for the PSNF group, mainly for the SPADI (Table 2). In relation to the Kappa values, the classifications of the PSNF group for the items of the DASH presented a moderate agreement with the literature, while the PHF group had a substantial agreement. The Kappa values were not obtained for the SPADI because the contingency table was developed only with two categories and one of them included only zero values.

**Table 2:** - Proportions of agreement and Kappa coefficient for the comparisons of the group of "professionals with high familiarity (PHF)" (n=4), and the group of "professionals with some or no familiarity (PSNF)" (n=19) with the literature

Literature	Proportions of Agreement (%)		Kappa (95% CI)	
	PSNF	PHF	PSNF	PHF
Roe et al.^30^	77%	92%	NC	NC
Drummond et al.^32^	80%	83%	0.46 (0.29 to 0.64)	0.62 (0.39 to 0.85)
Dixon et al.^31^	80%	87%	0.48 (0.24 to 0.72)	0.70 (0.45 to 0.96)

CI: 95% confidence interval; NC: value not calculated, because contingency table included a substantial proportion of zeros.

Table 3 shows that the PHF group diverged from the classification from the literature only in relation to item 11 in the pain domain: "When reaching for something on a high shelf with the affected arm?" The PSNF group could not relate the components of the ICF with items 9 ("How intense was the worst pain you had last week?"), 10 ("When did you lay on top of the affected arm?"), and 12 ("When you tried to touch the back of your neck with the affected arm?") of the pain domain.

**Table 3:** - Judgments of the group "professionals with high familiarity (PHF)", and the group of "professionals with some or no familiarity (PSNF)" for the items of The Shoulder Pain and Disability Index (SPADI-Brazil) according to International Classification of Functioning, Disability and Health (ICF) components of body function (F), body structure (S) and activity and participation (AP), and the comparisons with the literature.

	SPADI Items	PSNF			PHF			Classification of SPADI ^τ^		
		F	S	AP	F	S	AP	PSNF	PHF	Literature
		Mean	Mean	Mean	Mean	Mean	Mean			Roe et al.^30^
Items of Function	Item 1	–0.09	0.09	0.85*	–1.00*	–1.00*	1.00*	AP	AP	Body Function *- Sensorial and pain function (b2)* Activity and Participation *- Mobility (d4) and self-care (d5)*
	Item 2	0.15	0.01	0.68*	–0.98*	–0.50	1.00*	AP	AP	
	Item 3	0.05	–0.21	0.83*	–1.00*	–1.00*	1.00*	AP	AP	
	Item 4	–0.06	–0.03	0.88*	–100*	–1.00*	1.00*	AP	AP	
	Item 5	–0.21	–0.09	0.79*	–1.00*	–1.00*	1.00*	AP	AP	
	Item 6	–0.13	0.31	0.68*	–1.00*	–1.00*	1.00*	AP	AP	
	Item 7	–0.05	0.28	0.81*	–1.00*	–1.00*	1.00*	AP	AP	
	Item 8	0.05	0.21	0.79*	–1.00*	–1.00*	1.00*	AP	AP	
Items of Pain	Item 9	0.21	–0.2	–0.39	1.00*	–1.00*	–1.00*	NO	F	
	Item 10	0.11	0.14	0.03	1.00*	–1.00*	–0.50	NO	F	
	Item 11	0.29	0.18	0.60*	0.50	–0.98*	–0.50	AP	S	
	Item 12	0.07	0.15	0.07	1.00*	–1.00*	–0.50	NO	F	
	Item 13	0.17	0.32	0.43^¥^	1.00*	–1.00*	–0.50	AP	F	

NO: no classification. t indicates positive and negative values, respectively, associated or not associated with the ICF component. *p<0.05.

In relation to the DASH (Table 4), the PHF group did not associate four items of distinct domains with the components of the ICF, including items 21 ("Sexual Activities"), 22 ("Over the past week, at which point did your problem with the arm, shoulder or hand affected your normal activities with family, friends, neighbors or colleagues?"), 28 ("Difficulty in moving arm, shoulder or hand"), and 29 ("Over the past week, how difficult was it to sleep because of the pain in your arm, shoulder or hand?"). The PSNF group did not associate three items of the domain symptoms: items 26 ("Discomfort in the skin (pins and needles) on your arm, shoulder or hand"), 27 ("Weakness in the arm, shoulder or hand") and 28(see above).

**Table 4:** - Judgments of the group of "professionals with high familiarity (PHF)", and the group of "professionals with some or no familiarity (PSNF)" for the items of the Brazilian version of The Disabilities Arm, Shoulder and Hand (DASH) according to International Classification of Functioning, Disability and Health (ICF) components of body function (F), body structure (S) and activity and participation (AP), and comparisons with the literature.

	DASH Items	PSNF			PHF			Classification of DASH ^τ^			
		F	S	AP	F	S	AP	PSNF	PHF	Drummond et al.^32^	Dixon et al.^31^
Physical Function	Item 1	–0.29	–0.08	0.83*	–1.00*	–1.00*	1.00*	AP	AP	AP	A
	Item 2	–0.16	–0.11	0.83*	–0.63	–1.00*	0.95*	AP	AP	AP	A
	Item 3	0.14	–0.10	0.79*	–1.00*	–1.00*	–1.00*	AP	AP	AP	A
	Item 4	0.03	–0.03	0.87*	–1.00*	–1.00*	–1.00*	AP	AP	AP	A
	Item 5	–0.18	0.02	0.77*	–1.00*	–1.00*	–1.00*	AP	AP	AP	A
	Item 6	–0.03	–0.17	0.79*	–1.00*	–1.00*	–1.00*	AP	AP	AP	A
	Item 7	–0.04	–0.18	0.76*	–1.00*	–1.00*	–1.00*	AP	AP	AP	A
	Item 8	–0.21	–0.17	0.83*	–1.00*	–1.00*	–1.00*	AP	AP	AP	A
	Item 9	–0.09	–0.16	0.82*	–1.00*	–1.00*	–1.00*	AP	AP	AP	A
	Item 10	–0.24	–0.12	0.82*	–1.00*	–1.00*	–1.00*	AP	AP	AP	NO
	Item 11	–0.27	0.06	0.76*	–0.98	–1.00*	–1.00*	AP	AP	AP	A
	Item 12	–0.15	–0.04	0.80*	–1.00*	–1.00*	–1.00*	AP	AP	AP	A
	Item 13	–0.19	0.06	0.65*	–1.00*	–1.00*	–1.00*	AP	AP	AP	A
	Item 14	0.02	–0.08	0.76*	–1.00*	–1.00*	–1.00*	AP	AP	AP	A
	Item 15	–0.24	–0.07	0.86*	–1.00*	–1.00*	–1.00*	AP	AP	AP	A
	Item 16	–0.26	–0.01	0.88*	–1.00*	–1.00*	–1.00*	AP	AP	AP	A
	Item 17	–0.05	–0.02	0.84*	–1.00*	–1.00*	–1.00*	AP	AP	AP	AP
	Item 18	0.04	0.29	0.87*	–0.50	–1.00*	–1.00*	AP	AP	F and AP	AP
	Item 19	0.07	0.09	0.85*	–1.00*	–1.00*	–1.00*	AP	AP	F and AP	AP
	Item 20	0.05	–0.20	0.60*	–0.75	–1.00*	–1.00*	AP	AP	AP	AP
	Item 21	0.03	–0.22	0.80*	0.03	–1.00*	–0.05	AP	NO	AP	AP
Social Function	Item 22	0.30	0.05	0.55^£^	–1.00*	–0.50	0.50	AP	NO	AP	P
	Item 23	0.16	0.05	0.66*	–1.00*	–0.50	1.00*	AP	AP	AP	AP
Symptoms	Item 24	0.10	0.51^£^	–0.09	1.00*	–0.50	–0.75	S	F	F	SF
	Item 25	0.42^¥^	0.38	0.18	1.00*	–1.00*	–0.50	F	F	F	SF
	Item 26	0.14	0.25	–0.52*	1.00*	–0.48	–1.00*	NO	F	F	SF
	Item 27	0.36	0.34	–0.39	1.00*	–0.98*	–1.00*	NO	F	F	SF
	Item 28	0.44	0.17	0.39	0.58	–0.30	–0.60	NO	NO	F	SF
	Item 29	0.13	–0.01	0.47^£^	0.50	–0.50	–0.50	AP	NO	F	NO
Psychological Function	Item 30	0.49^¥^	–0.02	–0.07	0.45	–1.00*	–1.00*	F	NO	PF	NO

NO: no classification; A: activity; P: participation; SF: body structure and function; PF: personal factor. t indicates positive and negative values, respectively, associated or not associated with ICF component. *p<0.05.

Table 5 illustrates the judgments of the groups and presents the classifications of Dixon et al.^31^. Both PHF and PSNF groups related more frequently the items of the domain physical function of the DASH (items 1 to 16) to the component activity. The remaining items of the physical and social function domains (17 to 23), classified by the literature as activity-participation or participation, presented a frequency of judgment favoring participation, or a balance between activity and participation. The PSNF group presented a more random judgment pattern between activity and participation compared to the PHF group, which had a more consistent judgment.

**Table 5:** - Frequency of judgments for activity (A) and participation (P) for the items of the Brazilian version of The Disabilities Arm, Shoulder and Hand (DASH) for the group of "professionals with high familiarity (PHF)", and the group of "professionals with some or no familiarity (PSNF)".

DASH items	Items	PSNF	PHF	DASH by Dixon et al.^31^
Physical Function	Item 1	A (78.9%); P(21%)	A (100%)	A
	Item 2	A (78.9%); P(10.5%); AP(10.5%)	A (100%)	A
	Item 3	A (63.2%); P(21%); AP(5.3%)	A (100%)	A
	Item 4	A(73.7%); P(26.3%)	A (100%)	A
	Item 5	A (52.6%) ; P(26.3%); AP(15.8%)	A (100%)	A
	Item 6	A (78.9%); P(10.5%); AP(5.3%)	A (100%)	A
	Item 7	A (73.7%); P(15.8%); AP(5.3%)	A (100%)	A
	Item 8	A (57.9%); P(36.8%); AP(5.3%)	A(50%); P(50%)	A
	Item 9	A (57.9%); P(26.3%); AP(15.8%)	A (100%)	A
	Item 10	A (73.7%); P(21%)	A (100%)	NO
	Item 11	A (84.2%); P(10.5%)	A (100%)	A
	Item 12	A (73.7%); P(21%)	A (100%)	A
	Item 13	A (63.2%); P(21%); AP(5.3%)	A (100%)	A
	Item 14	A (68.4%); P(21%); AP(5.3%)	A (100%)	A
	Item 15	A (73.7%); P(15.8%); AP(10.5%)	A (100%)	A
	Item 16	A (78.9%); P(21%)	A (100%)	A
	Item 17	A (47.4%); P(42.1%)	A(25%); P(75%)	AP
	Item 18	A (68.4%); P(31.6%)	A(25%); P(75%)	AP
	Item 19	A (52.6%); P(42.1%); AP(5.3%)	A(50%); P(50%)	AP
	Item 20	A (36.8%); P(47.4%); AP(5.3%)	A (100%)	AP
	Item 21	A (31.6%); P(52.6%); AP(15.8%)	NO	AP
Social Function	Item 22	A (15.8%); P(63.2%)	NO	P
	Item 23	A (31.6%); P(52.6%); AP(5.3%)	P(75%); AP(25%)	AP
Symptoms	Items 24-28	*They were not discriminated in table because they are not classified by Dixon et al.* ^31^ *as activity or participation*		
	Item 29	A (31.6%); P(36.8%); AP(5.3%)	NO	NO

NO: no classification.

## Discussion

In agreement with the literature, the data showed that health professionals who research or treat upper limb injuries acknowledge the components of the ICF for most of the items of the two shoulder questionnaires studied, independent of their level of familiarity with the ICF. However, the classification of the DASH questionnaire obtained from the PSNF participants agreed moderately with the literature, while the PHF group agreed substantially, supporting our initial hypothesis.

The classifications for the SPADI were compared with the study by Roe et al^30^. They considered body function and activity-participation the correct choices for the classifications. Although the PHF and PSNF groups classified most of the items of the SPADI according to the literature, the content discriminant validity method showed that the PSNF group failed to define any of the components for the three items of pain, while the PHF group missed only one item. It was not possible to confirm that the definition of the ICF^1^ for pain was insufficient for a proper classification of the items for pain since the Brazilian version of the SPADI has not been tested for content validity. However, one could consider such a possibility since the SPADI-Brazil has satisfactory construct validity, with a high (-0.78) to moderate (0.68) correlation of its pain domain with the pain domain from two other functional questionnaires - Penn Shoulder Score (PSS) and Short-Form 36 (SF-36)^46^, respectively. Besides, all the test measurement properties of the SPADI-Brazil were similar to the original version in English, therefore it is likely to occur with the content validity, which showed in the original version that the SPADI has no validity problems for the pain items^47^^,^ 48.

It is important to highlight the need for some specific training instead of only presenting the definitions of the components of the ICF. Therefore, health professionals could recognize the components of the pain items of the SPADI, since the questionnaire evaluates pain of the patient in the context of activities of daily living. This would give health professionals, who had little or no familiarity with the ICF, the chance to associate these items to the activity-participation component. However, pain is described in Chapter 2 (Sensory Function and Pain)^1^ of the International Classification of Functioning, Disability and Health (ICF) under the body function component, and the health professionals of the PHF group who worked and/or performed research using the ICF had no difficulty identifying this classification.

The literature describes two studies that related to the items of the DASH with the components of the ICF^31^^,^ 32. Drummond et al.^32^ classified each item of the DASH as belonging to the components - body function, body structure and activity-participation. Dixon et al.^31^ however, considered the components function and body structure together and the components activity and participation separately.

In this study, the classifications of the PSNF and PHF groups agreed with more than 80% of the literature, considering the findings of Dixon et al.^31^ and Drummond et al.^32^. Moreover, the Kappa values were moderate to substantial, indicating that the components of the ICF might easily relate to the DASH items. However, disagreements in classifications occurred for the items 21, 22 and 26 to 29 from the symptom domain.

All of the items from the DASH in which the ratings from the health professionals disagreed with the two classifications adopted by the literature^31^^,^ 32 (items 21, 22 and 26 to 29), had poor content validity results. Franchignoni et al.^49^ recognized some problems with the validity of the English version of the DASH questionnaire for items 21 and 26. In the Brazilian version, the factorial analysis showed that items 26, 27, 28 and 29 from the symptoms domain belonged to a different domain^50^. Therefore, it is suggested that the content validity was poorly established for these items and that it may have interfered with the ability of the health professionals from the present study to define which component of the ICF the item was related to.

The PHF group agreed relatively more in their ratings regarding the ability to distinguish activity and participation compared to the PSNF group. Thus, it appears that beyond the correct definitions of these components, other strategies were necessary to clarify that the activity component was related to the activities of daily living and the participation to social activities^1^. This differentiation is important for clinicians, since different questionnaires used in the clinical setting very often include activity components^30^^,^ 31, and it is important for the health professionals to complement the evaluation with information from the participation domain.

Study limitations have occurred and are related to the sample size of the PSNF group, although the power analysis was acceptable. The PSNF group was a sample of convenience and some losses occurred. Thus, the data from the PSNF group must be extrapolated only for health professionals with similar characteristics, i.e., those who usually do not work with the ICF, or are untrained and have a limited knowledge of the ICF, or less than five points on the numerical rating scale of 10. In addition, the participants were not asked about their prior knowledge of the DASH and SPADI questionnaires, which may have lead to difficulty in the recognition of the items.

Finally, the strongest aspect of this study is the initiative to explore whether health professionals, with different levels of familiarity, are able to identify ICF components in questionnaires that have been validated for the Brazilian language and that are relatively well-known to clinicians. Thus, although the results are exploratory, it was possible to identify that professionals could relate the ICF components with most items on the questionnaires, regardless of the level of their familiarity with the ICF classification. In addition, it was also observed that the concept of pain was the most difficult to categorize. Therefore, the relation of pain with body function component of ICF deserves more attention when trying to correlate the ICF with functional outcome questionnaires.
